# Roles of ZEB1 and ZEB2 in E‐cadherin expression and cell aggressiveness in head and neck cancer

**DOI:** 10.1111/gtc.13167

**Published:** 2024-10-03

**Authors:** Arisa Kinouchi, Takahiro Jubashi, Rikito Tatsuno, Jiro Ichikawa, Kaname Sakamoto, Daiju Sakurai, Tomonori Kawasaki, Hiroki Ishii, Keiji Miyazawa, Masao Saitoh

**Affiliations:** ^1^ Department of Biochemistry University of Yamanashi Chuo Yamanashi Japan; ^2^ Department of Otolaryngology, Head and Neck Surgery University of Yamanashi Chuo Yamanashi Japan; ^3^ Department of Orthopaedic Surgery University of Yamanashi Chuo Yamanashi Japan; ^4^ Department of Pathology Saitama Medical University International Medical Center Saitama Japan; ^5^ Center for Medical Education and Sciences, Graduate School of Medicine University of Yamanashi Chuo Yamanashi Japan

**Keywords:** EMT, head and neck squamous cell carcinoma, ZEB1, ZEB2

## Abstract

Zinc finger E‐box binding homeobox 1 (ZEB1) has been identified as a key factor in cancer cell differentiation and metastasis, and has been well studied in the field of cancer cell biology. ZEB2 has a highly similar conformation to ZEB1, but its role in head and neck squamous cell carcinoma (HNSCC) cells is not fully understood. Here, we separately overexpressed ZEB1 and ZEB2 in C57BL/6 mouse oral cancer (MOC) cells and investigated their cellular characteristics, including E‐cadherin levels, motile properties, chemoresistance, and metastatic ability in immunocompetent mice. Both ZEB1 and ZEB2 overexpression reduced epithelial traits and converted cells to an aggressive phenotype. Surprisingly, ZEB1 overexpression increased the endogenous level of ZEB2 in MOC cells, and vice versa. The molecular mechanisms underlying these findings remain unclear. However, the in vitro anchorage‐independent growth of MOC cells overexpressing ZEB2 was considerably greater than that of MOC cells overexpressing ZEB1. These findings suggest that ZEB2, like ZEB1, has the ability to induce the differentiation of cancer cells into those with highly aggressive traits.

## Graphical abstract

A two‐well silicone insert for cell co‐cultivation was used because this method makes it possible to obtain photos of the two cell types simultaneously after immunostaining, with subsequent comparison of their protein expression levels. After MOC1 and MOC1‐ZEB1 cells were seeded separately in two‐well silicone inserts, actin cytoskeleton reorganization was visualized by tetramethyl rhodamine isothiocyanate (TRITC)‐phalloidin staining.

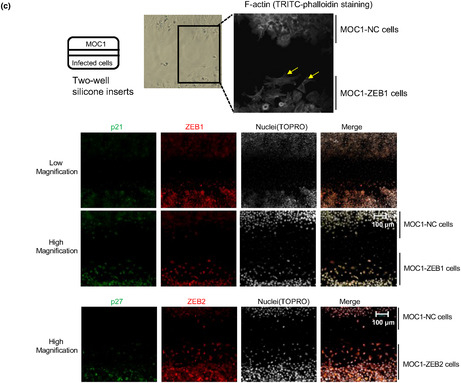

## 1 INTRODUCTION

Head and neck cancers arise from the mucosal epithelium in the oral cavity, pharynx, and larynx, and are usually squamous cell carcinoma. At their first hospital visit, most patients with head and neck squamous cell carcinoma (HNSCC) already have local metastases in regional lymph nodes, without major functional impairment. Thus, the overall 5‐year survival rate remains approximately 40% despite recent advances in multimodal therapy (Okano, 2024).

Cancer cell invasion, metastasis, and chemoresistance to anti‐cancer drugs all require cell differentiation through the transcriptional regulation of numerous genes (Pawlicka et al., 2024). One important process in this regard is known as the epithelial‐mesenchymal transition (EMT), which is triggered by several transcription factors (hereafter referred to as EMT‐TFs), mainly those belonging to the zinc‐finger E‐box binding homeobox (ZEB), Snail, and basic helix‐loop‐helix (bHLH) families of proteins. The ZEB family consists of two‐handed zinc‐finger transcription factors, including ZEB1 and ZEB2. The Snail family comprises the zinc‐finger transcription factors Snail (Snail1) and Slug (Snail2), while bHLH family proteins include the E26 transformation‐specific (ETS) family and the Twist family. Among bHLH family proteins, ETS1, ETS2, ELF3 (E74‐Like Factor 3, a.k.a. ESE1), and EHF (ETS homologous factor, a.k.a. ESE3) play crucial roles in the EMT program and differentiation in various cancer cells (Saitoh, 2023; Suzuki et al., 2021). Recently, we found that ETS1 and ETS2 promote EMT in HNSCC cells, whereas EHF and ELF3 inhibit it (Sakamoto et al., 2021; Sinh et al., 2017). Importantly, mutations within the conserved ETS domain in EHF and ELF3 abolish the ability of these proteins to inhibit EMT, and this subsequently promotes tumor growth and invasion in vivo, leading to a dominant negative fashion on EMT and cancer cell differentiation in vivo (Sakamoto et al., 2021).

Among various types of cancers, human breast cancer cells and human HNSCC cells have been well characterized in EMT studies, and show a positive correlation between the expression of ZEB1/2 (ZEB1 and ZEB2) and aggressive cancer cell phenotypes. Further, Snail has been associated with aggressive phenotypes such as chemoresistance in human pancreatic cancer (Zheng et al., 2015), but not with the mesenchymal trait in human breast cancer cells and human HNSCC cells (Fukagawa et al., 2015; Horiguchi et al., 2012; Sakamoto et al., 2021). MOC cells are generated from oral cancer in C57BL/6 mice using 7, 12‐dimethylbenz(a) anthracene (DMBA), with similar characteristics to human HNSCC, and are divided into two subclones, MOC1 and MOC2. MOC1 cells are derived from a mucosal lip lesion, and generate indolent tumors with increased expression of programmed death ligand 1 (PD‐L1) and MHC class I and secretion of interferon‐γ, and with increased CD8+ T‐cell infiltration into the tumor microenvironment. Thus, MOC1‐generated tumors are responsive to immunotherapy, often referred to as immunologically “hot” tumors. By contrast, MOC2 cells are generated from a floor of mouth mass, and generate aggressive tumors with a subset of *K‐Ras* mutation and low frequency of genetic alterations. MOC2‐generated tumors have decreased expression of MHC class I, and are associated with FOXP3^+^CD4^+^ regulatory T‐cell infiltration, often referred to as immunologically “cold” tumors, unresponsive to immunotherapy (Judd et al., 2012; Kono et al., 2022). However, the roles of ZEB1/2 in promoting EMT and MOC cell aggressiveness remain unclear.

In this study, we found that compared with MOC2 cells, MOC1 cells showed lower expression of ZEB1/2 and higher levels of E‐cadherin. Ectopic expression of either ZEB1 or ZEB2 in MOC1 cells repressed E‐cadherin levels, whereas Snail overexpression did not. Importantly, ectopic expression of ZEB1/2 resulted in mutual promotion of each other's expression, that is, overexpression of ZEB1 upregulated endogenous ZEB2 expression, and vice versa, and E‐cadherin was downregulation in both cases. In in vivo studies, we found that MOC1 cells overexpressing either ZEB1 or ZEB2 had aggressive phenotypes and were lethal in mice. Although the mechanisms underlying these findings remain unclear, ZEB1/2 are crucial factors for determining lethal HNSCC phenotypes in mice.

## 2 RESULTS

### 2.1 Overexpression of ZEB1 or ZEB2 in C57BL/6 MOC cells

**FIGURE 1:**
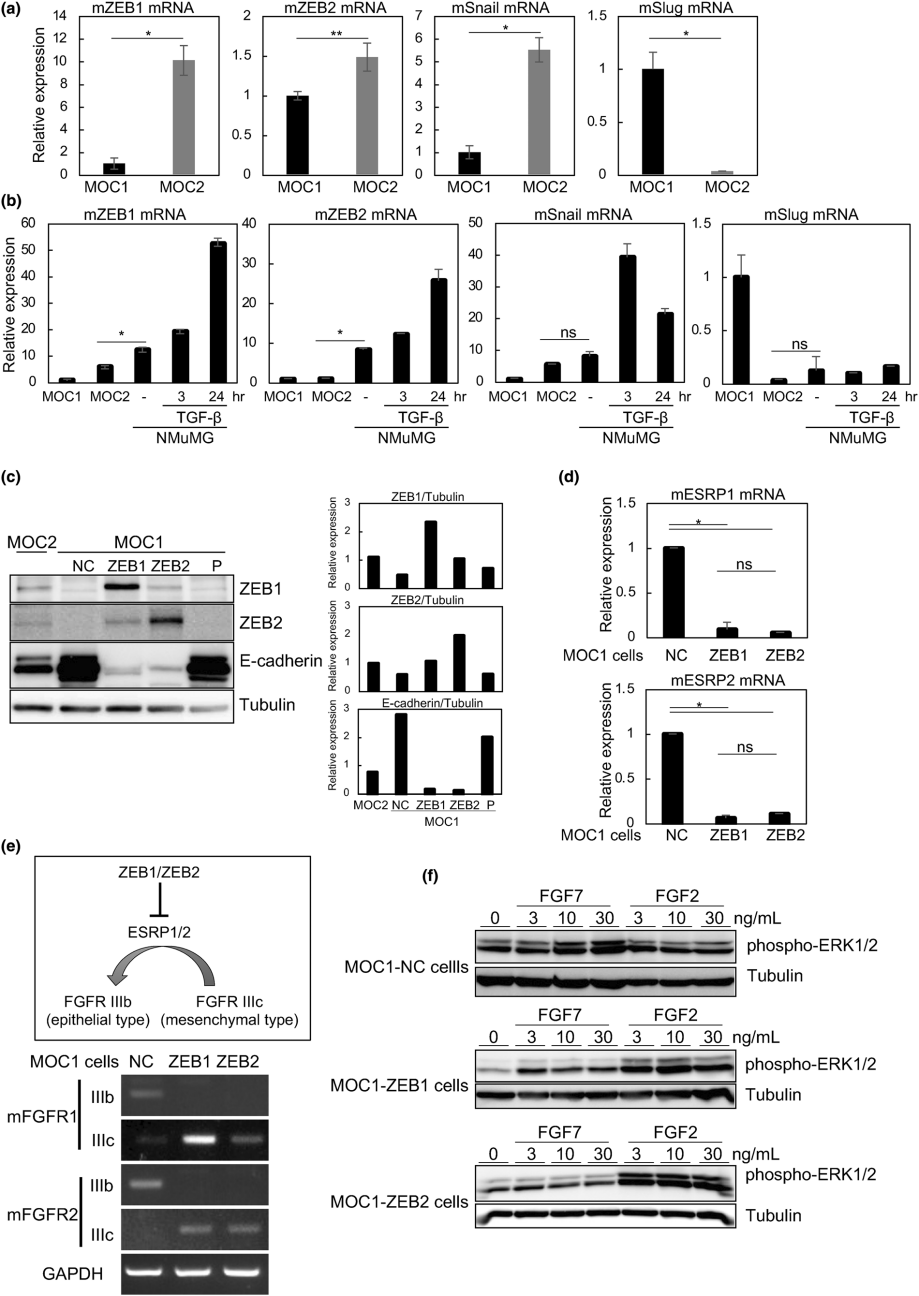
Comparisons between C57BL/6 MOC1 and MOC2 cells. (a, b) mRNA levels of representative EMT‐TFs were determined by RT‐qPCR. Upon TGF‐β stimulation for the indicated times, normal mouse mammary epithelial NMuMG cells were used as controls to assess the expression levels of EMT‐TFs. The ratio of each mRNA to *TBP* in MOC1 cells was defined as 1. (c) Immunoblotting with the indicated antibodies was performed after infection with lentiviruses carrying negative control (NC), ZEB1, or ZEB2. The ratio of ZEB1, ZEB2, or E‐cadherin to α‐tubulin was validated by densitometric analysis and shown at the right panels. The ratio of each protein to α‐tubulin in MOC2 cells was indicated as 1. P, parental MOC1 cells (non‐infected cells). (d, e) Expression levels of ESRP1 and ESRP2 were determined by RT‐qPCR (d). Expression levels of the alternately spliced forms of FGFR1 and FGFR2 were examined by conventional PCR (e). A schematic illustration of the relationship between ZEB1/2, ESRP1/2, and FGFRs is shown at the top (e). NC, negative control (d, e). (f) ERK1/2 phosphorylation (phospho‐ERK1/2) in response to FGF2 or FGF7 was examined by immunoblot analysis. Cells were stimulated with the indicated concentrations of FGF2 or FGF7 in the presence of heparin for 30 min. α‐tubulin was used as a loading control (c, f). *p* values were determined by Student's *t*‐test. *p < .01, **p < .05. ns, not significant. Each value represents the mean ± SD of duplicate measurements from a representative experiment.

We previously reported that ZEB1/2 are positively correlated with the expression of mesenchymal marker proteins and the aggressiveness of various types of cancer cells, including those from human HNSCC and human breast cancer (Horiguchi et al., 2012; Sakamoto et al., 2021). In addition, ZEB1/2 silencing resulted in less aggressive phenotypes in human HNSCC (Osada et al., 2019). To determine the roles of ZEB1/2 in mouse HNSCC in vivo, we used C57BL/6 MOC cells in this study. MOC1 cells form localized tumors with well‐differentiated features, whereas MOC2 cells form metastatic, poorly differentiated tumors (Judd et al., 2012). We first determined the mRNA levels of representative EMT‐TFs in MOC cells (Figure 1a). ZEB1 and Snail were dramatically downregulated in MOC1 cells compared with MOC2 cells, while Slug mRNA levels were extremely high in MOC1 cells. The mRNA levels of ZEB2 were slightly lower in MOC1 cells than in MOC2 cells, while its protein levels were significantly lower in MOC1 cells (Figure 1c). The mRNA levels of Twist family proteins and ETS family proteins, including ETS1, ETS2, ELF1, and EHF, were not significantly different between MOC1 and MOC2 cells (data not shown).

EMT induced by transforming growth factor‐β (TGF‐β) was previously investigated in normal mouse mammary epithelial NMuMG cells (Shirakihara et al., 2011). In the present study, basal levels of ZEB1/2 were considerably lower in MOC2 cells than in NMuMG cells, and basal levels of Snail were similar between MOC2 and NMuMG cells (Figure 1b). Conversely, Slug expression levels were much higher in MOC1 cells than in NMuMG cells (Figure 1b). It is well known that TGF‐β suppresses E‐cadherin and induces EMT in NMuMG cells, and both processes are highly dependent on ZEB1/2 upregulation by TGF‐β (Shirakihara et al., 2011). In the current study, the expression levels of ZEB1/2 and Snail were significantly higher in NMuMG cells stimulated with TGF‐β than in MOC1 and MOC2 cells. Thus, we measured E‐cadherin levels in MOC1 cells after infection with lentiviruses carrying ZEB1 (MOC1‐ZEB1 cells), ZEB2 (MOC1‐ZEB2 cells), Snail, or a negative control (hereafter referred to as MOC1‐NC cells). The expression levels of ZEB1/2 were determined by immunoblot analysis (Figure 1c). Surprisingly, ZEB1 and ZEB2 mutually increased each other's expression, that is, ectopic expression of ZEB1 increased endogenous ZEB2 expression, and vice versa (Figure 1c). In addition, E‐cadherin was almost undetectable in MOC1‐ZEB1 and MOC1‐ZEB2 cells. On the other hand, infection with a lentivirus carrying cDNA against Snail failed to alter the expression of E‐cadherin (Figure S1a).

ZEB1/2 reportedly bind directly to the promoter regions of epithelial‐splicing regulatory proteins 1 and 2 (ESRP1/2), and transcriptionally suppress these proteins (Horiguchi et al., 2012). Both ESRP1 and ESRP2 activate the splicing of exon IIIb and silence that of exon IIIc in fibroblast growth factor receptor (FGFR) genes (Warzecha et al., 2009). MOC1‐NC cells exhibited the epithelial isoform (IIIb) of FGFR1 and FGFR2. After overexpression of ZEB1 or ZEB2, ESRP1/2 were downregulated (Figure 1d), and the isoforms of FGFR1 and FGFR2 changed from IIIb to IIIc (Figure 1e). Fibroblast growth factor (FGF)‐2 and FGF4 bind preferentially to the IIIc isoforms and activate extracellular signal‐regulated kinase (ERK) 1 and 2 pathways, while FGF7 and FGF10 bind exclusively to the IIIb isoforms. In MOC1‐NC cells, FGF7, but not FGF2, activated ERK1 and 2 (ERK1/2), whereas ERK1/2 phosphorylation in MOC1‐ZEB1 and MOC1‐ZEB2 cells was observed in response to FGF2, but not FGF7 (Figure 1f). Thus, epithelial‐type MOC1 cells differentiate into EMT phenotype cells by overexpression of ZEB1/2.

### 2.2 Roles of ZEB1/2 in E‐cadherin repression in mouse HNSCC cells

**FIGURE 2:**
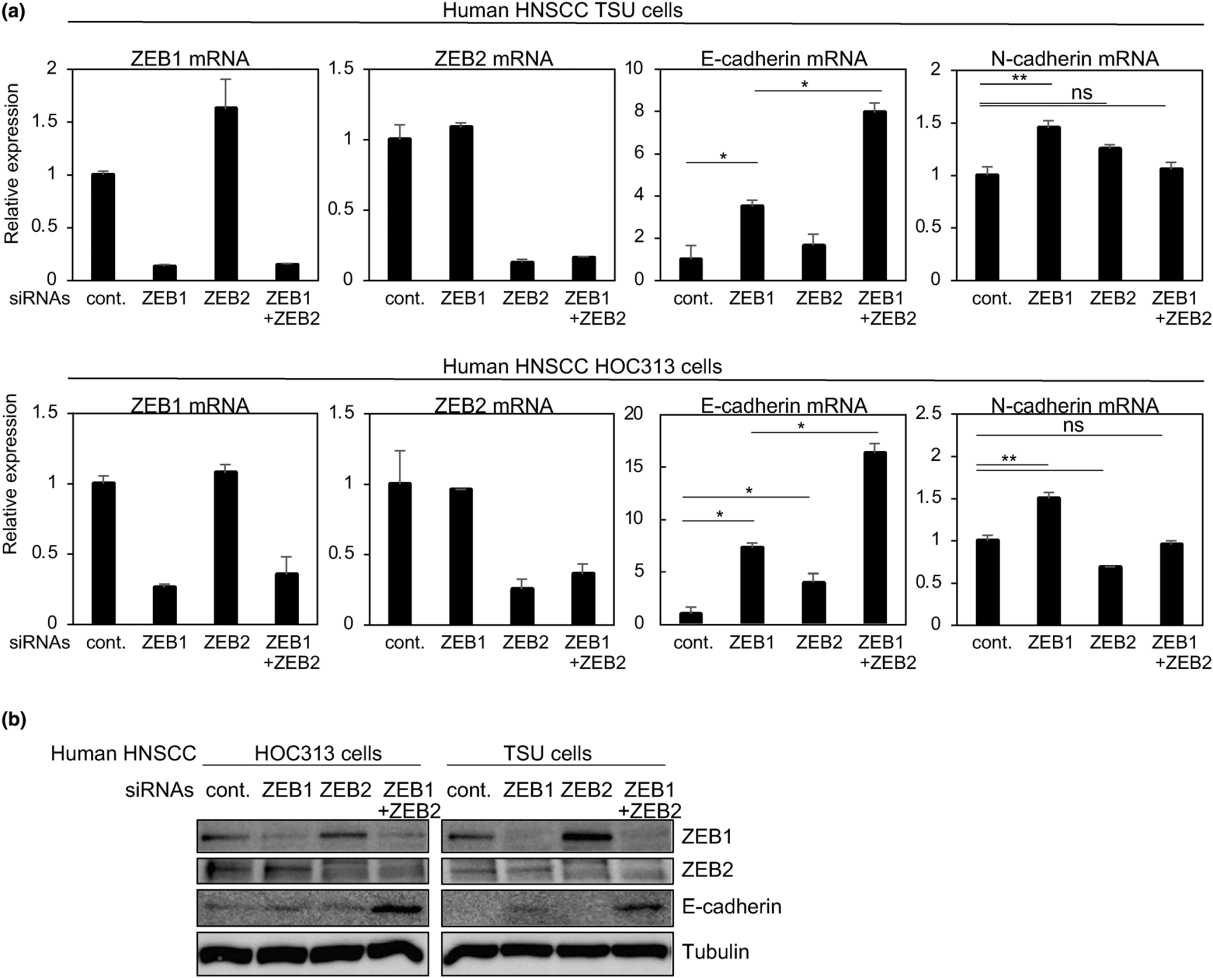
Knockdown of ZEB1/2 in human HNSCC cells. (a, b) After transfection with siRNAs, the expression levels of E‐cadherin and N‐cadherin in human HNSCC TSU and HOC313 cells were determined by RT‐qPCR (a) and immunoblot analysis (b). cont., control siRNA (a and b). α‐tubulin was used as a loading control (b). P values were determined by Tukey's multiple comparison test. *p < .01, **p < .05. ns, not significant. Each value represents the mean ± SD of duplicate measurements from a representative experiment.

MOC1‐ZEB1 and MOC1‐ZEB2 cells exhibited dramatically reduced E‐cadherin levels. However, MOC1‐ZEB1 cells with ectopic expression of ZEB1 showed high expression of endogenous ZEB2, and vice versa (see Figure 1c). To evaluate whether E‐cadherin repression was due to ZEB1, ZEB2, or both, we sought to establish knockout MOC cells using CRISPR/Cas9 techniques with available gRNAs against the *Zeb1* and *Zeb2* genes, and to generate knockdown MOC cells by introducing short hairpin RNAs (shRNAs) targeting mouse Zeb1 and Zeb2. Unfortunately, we could not establish MOC cells in which endogenous Zeb1 and Zeb2 were knocked out or knocked down (data not shown). Thus, instead of MOC cells, we used two types of human HNSCC cells, specifically TSU and HOC313 cells, for further analyses, because both cell types express low levels of E‐cadherin and high levels of ZEB1/2 (Sakamoto et al., 2021). The efficacy of the small interfering RNAs (siRNAs) targeting human ZEB1 and ZEB2 in both cell types was assessed by reverse transcription polymerase chain reaction (RT‐qPCR) and immunoblot analyses (Figure 2a,b). E‐cadherin levels were increased by transfection with both ZEB1 and ZEB2 siRNAs, but not either alone. These findings suggest that expression levels of E‐cadherin are regulated by ZEB1 and ZEB2.

### 2.3 Aggressive phenotypes of MOC1 cells overexpressing ZEB1 or ZEB2

**FIGURE 3:**
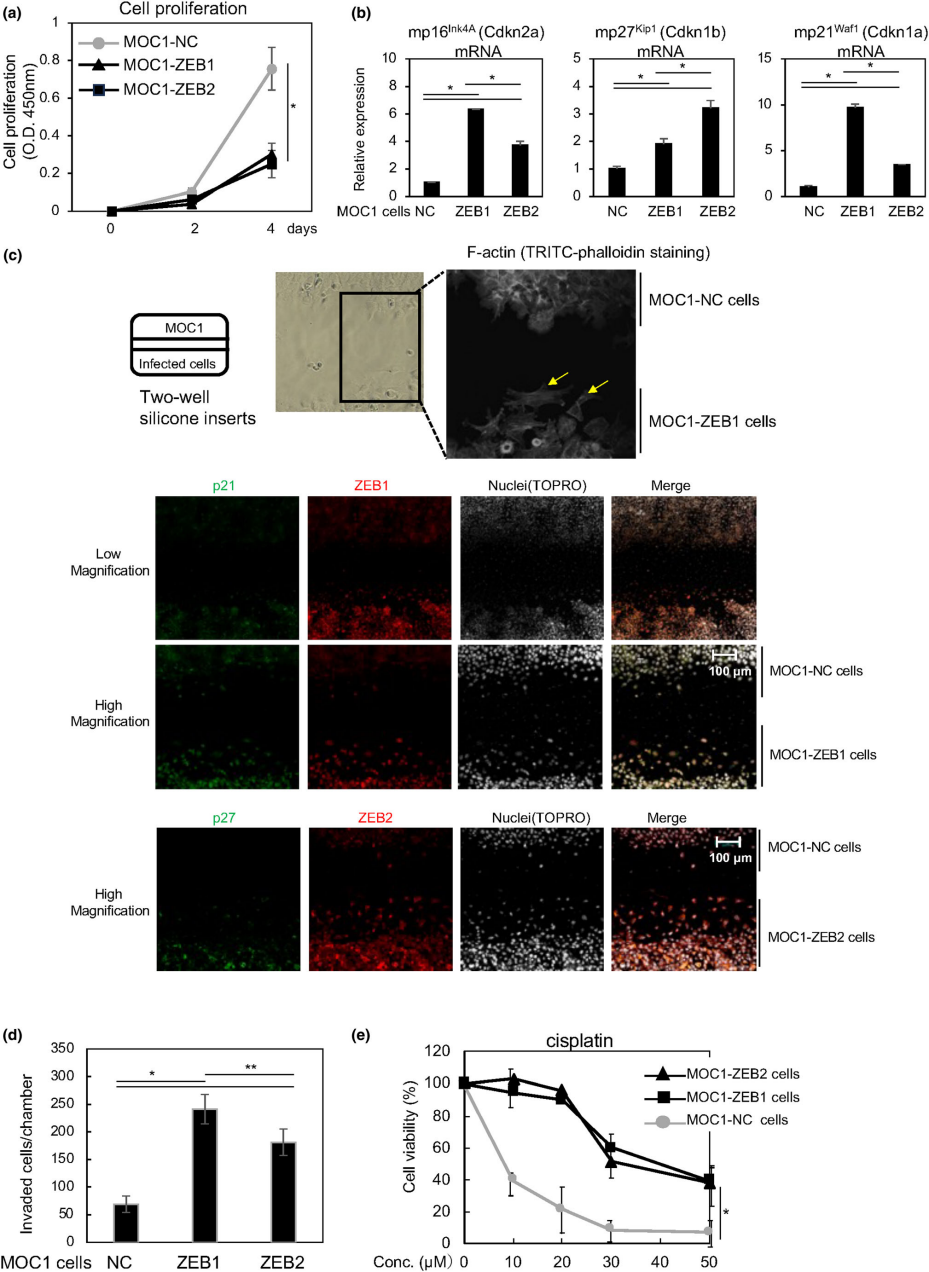
Inhibition of cell proliferation by ZEB1/2. (a) Cell proliferation rates were determined with an MTT assay. Each value represents the mean ± SD of triplicate measurements from a representative experiment. Similar results were obtained in two independent experiments. (b) mRNA levels of representative CDK inhibitors were determined by RT‐qPCR. Each value represents the mean ± SD of duplicate measurements from a representative experiment. NC, negative control. (c) Two types of cells were seeded on a two‐well silicone insert. Approximately 24 h later, the insert was removed from the plate. After gap creation, the cells were monitored and visualized by phase contrast microscopy, followed by TRITC‐phalloidin staining or immunohistochemical analysis with the indicated antibodies. Yellow arrows show typical F‐Actin stress fiber formation in MOC1‐ZEB1 cells. (d) Cells were seeded on cell culture inserts. After 12 h, the cells that had migrated to the opposite side were stained and quantified. Each value represents the mean ± SD of triplicate measurements from a representative experiment. Similar results were obtained in two independent experiments. NC, negative control. (e) Chemoresistance assay in response to the indicated concentrations (Conc.) of cisplatin. Each value represents the mean ± SD of quadruple measurements from a representative experiment. Similar results were obtained in two independent experiments. P values were determined by Student's *t*‐test (a and e) or by Tukey's multiple comparison test (b, d). *p < .01, **p < .05.

We examined the in vitro cell proliferation of MOC1, MOC1‐ZEB1, and MOC1‐ZEB2 cells (Figure 3a). Overexpression of either ZEB1 or ZEB2 reduced the cell proliferation rate by approximately 50% on day 4. In addition, the expression levels of cyclin‐dependent kinase (CDK) inhibitors such as p16^INK4A^, p27^KIP1^, and p21^WAF1^ were elevated in both MOC1‐ZEB1 and MOC1‐ZEB2 cells (Figure 3b). Next, we utilized a two‐well silicone insert for cell co‐cultivation because this method makes it possible to obtain photos of the two cell types simultaneously after immunostaining, with subsequent comparison of their protein expression levels (Figure 3c, left). After MOC1 and MOC1‐ZEB1 cells were seeded separately in two‐well silicone inserts, actin cytoskeleton reorganization was visualized by tetramethyl rhodamine isothiocyanate (TRITC)‐phalloidin staining (Figure 3c, right). Overexpression of ZEB1 led to considerable changes in cell morphology and actin fiber formation, which are typical of fibroblastic differentiation (Figure 3c, right). Immunohistochemistry analysis using a two‐well silicone insert showed that protein levels of the CDK inhibitors p21^WAF1^ and p27^KIP1^ were slightly upregulated in MOC1‐ZEB1 and MOC1‐ZEB2 cells, respectively, compared with MOC1‐NC cells. A Boyden chamber assay using inserts coated with type I collagen gel showed that MOC1‐ZEB1 and MOC1‐ZEB2 cells exhibited greater motility than MOC1‐NC cells (Figure 3d). An analysis of anti‐cancer drug sensitivity indicated that sensitivity to cisplatin was significantly decreased in MOC1‐ZEB1 and MOC1‐ZEB2 cells (Figure 3e). Together, these in vitro experiments suggest that MOC1 cells overexpressing either ZEB1 or ZEB2 acquire EMT phenotypes and aggressiveness.

### 2.4 Tumor‐promoting effects of ZEB1/2 in vivo

**FIGURE 4:**
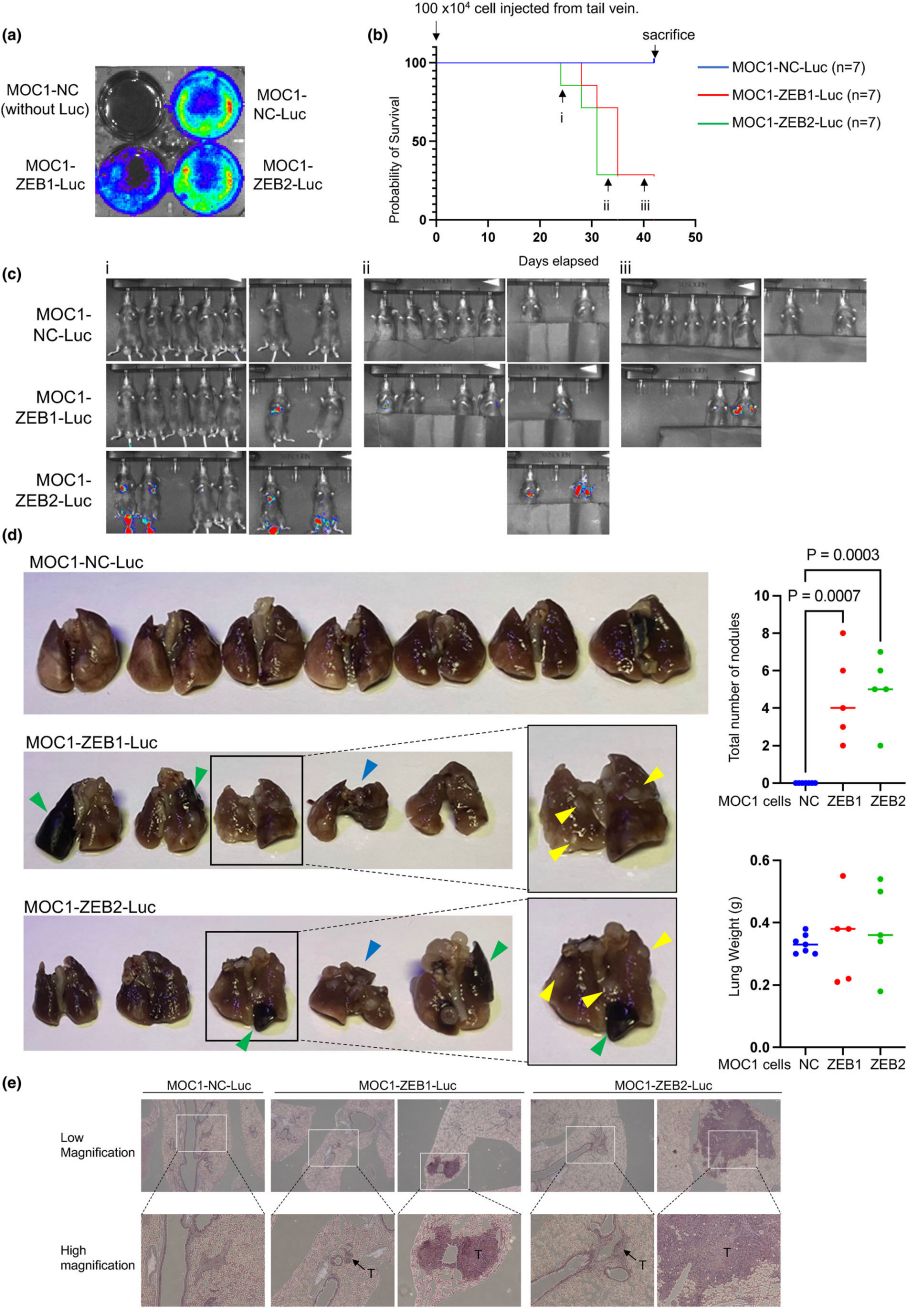
Tumor‐promoting effects of ZEB1/2 in immunocompetent mice (a) The luciferase activity of cells infected with lentiviruses carrying the luciferase reporter (Luc) was measured with the IVIS Lumina imaging system. (b–d). After the cells were injected intravenously into the tail veins of syngeneic C57BL/6 mice (*n* = 7), survival rates were analyzed using the Kaplan–Meier method (b). Beginning 14 days after the injection, the emission intensity was measured using the IVIS Lumina imaging system about once every 3 days. Representative image data at 24 days (i), 33 days (ii) and 40 days (iii) are shown (c). Lungs were extracted from mice that died from cancer and from mice that were sacrificed due to severe debility, followed by macroscopic analysis (left in d), determination of the total number of nodules, and measurement of lung weight (right in d). Yellow arrowheads show nodules that were observed macroscopically. Green and blue arrowheads show abnormalities due to massive hemorrhage and consolidation, respectively (d). P values were determined by Tukey's multiple comparison test (d). Typical histological findings following HE staining are shown (f). T, tumor.

Since the aforementioned in vitro experiments showed that MOC1‐ZEB1 and MOC1‐ZEB2 cells developed aggressive phenotypes, we further evaluated their properties using an experimental model of pulmonary metastasis in mice. First, MOC1‐NC, MOC1‐ZEB1, and MOC1‐ZEB2 cells were infected simultaneously with lentiviruses carrying a luciferase reporter. We measured the in vitro luciferase activity of these three cell types at the same time. The luciferase activity of MOC1‐ZEB1 cells was significantly lower than that of the other two cell types (Figure 4a). After the cells were injected into the tail veins of syngeneic C57BL/6 mice, survival rates and luciferase activity were analyzed using the Kaplan–Meier method and an IVIS Lumina imaging system, respectively (Figure 4b,c). It has been reported that MOC1 cells do not metastasize in mice (Judd et al., 2012), whereas overexpression of ZEB1/2 readily caused metastases, at least in the lungs, and is lethal in mice. Lungs were extracted from mice that died from cancer and from mice that were sacrificed due to severe debility, followed by macroscopic analysis and hematoxylin and eosin (HE) staining after embedding (Figure 4d,e). In macroscopic analysis, tumors were never detected in the lungs of mice injected with MOC‐NC cells, whereas the lungs of mice injected with MOC1‐ZEB1 or MOC1‐ZEB2 cells showed several nodules with severe morphological abnormalities such as massive pulmonary hemorrhage and consolidation (Figure 4d). HE staining also demonstrated typical tumors in the lungs of mice injected with both MOC1‐ZEB1 and MOC1‐ZEB2 cells. Therefore, these findings suggest that ZEB1/2 both contribute to the aggressiveness of HNSCC in immunocompetent mice.

## 3 DISCUSSION

ZEB1 is one of the most extensively studied molecules in the field of EMT and cancer cell differentiation, while ZEB2 has not been researched in depth. The first objective of this study was to compare the differences in function between ZEB1 and ZEB2. In vitro and in vivo experiments showed similar functions of ZEB1 and ZEB2, that is, both inhibited cell proliferation, likely by inducing CDK inhibitors, and both promoted anti‐cancer drug resistance and motile properties. Importantly, we also analyzed anchorage‐independent growth by cultivating cells in soft agar. ZEB2 promoted proliferation in soft agar much more than ZEB1 and control (Figure S1b). Although only a few in vitro studies have examined the differences between ZEB1 and ZEB2, it was reported that ZEB1 synergizes with Smad proteins to accelerate TGF‐β signaling, whereas ZEB2 has the opposite effect (Postigo, 2003; Verschueren et al., 1999). Since TGF‐β promotes anchorage‐independent growth of cancer cells, it remains unclear why ZEB2 promotes proliferation in soft agar to a greater extent than ZEB1.

Regarding pathological findings, it is widely known that infiltrating cancer cells and cancer cells at the invasion front are growth arrested. In addition, in an elegant series of genetic experiments in *Caenorhabditis elegans*, Matus et al. clearly demonstrated that invasion required cell cycle arrest and that G1 arrest triggered changes in the differentiation state with the expression of invasion markers (Matus et al., 2015). They also showed that the nuclear receptor nhr‐67 caused G1 arrest in part by regulating the CDK inhibitor cki‐1. The loss of nhr‐67 resulted in non‐invasive, mitotic cells that failed to express matrix metalloproteinases and lacked invadopodia. Importantly, the ectopic expression of cki‐1 was sufficient to force G1 arrest and rescue cell invasion in nhr‐67‐deficient cells. The *nhr‐67* gene of *Caenorhabditis elegans* encodes the ortholog of vertebrate *Tlx* genes, and the *cki‐1* gene is a member of the Cip/Kip family of CDK inhibitors. In this study, we found that the CDK inhibitor p27^KIP1^ was transcriptionally promoted by ZEB1 (Figure 3a,b). Thus, it is possible that ZEB1/2 interact with *TLX* in HNSCC to promote the expression of CDK inhibitors.

There has recently been a significant amount of research on cancer immunity. The microRNA‐200 (miR‐200) family directly targets ZEB1, which is known to repress miR‐200 transcription (Gregory et al., 2008). In addition, miR‐200 directly binds to the 3′ UTR of programmed death‐ligand 1 (PD‐L1), a ligand that binds to the receptor programmed death‐1 (PD‐1) (Chen et al., 2014). Thus, cancer cells with high levels of ZEB1 exhibit aggressiveness and cause immunosuppression by inducing exhaustion of CD8^+^ T cells. On the other hand, it is uncertain if ZEB2 regulates PD‐L1 expression in cancer. In this study, we used RT‐qPCR to analyze the expression of PD‐L1 in MOC1 cells that overexpressed ZEB1 or ZEB2, and found that neither protein's overexpression significantly changed the mRNA levels of PD‐L1. We are currently using the mouse specimens from this study to perform immunohistochemical evaluations of the expression of PD‐L1‐positive tumor cells and the density of immune cells expressing CD8 (a marker of cytotoxic T cells), CD16 (a marker of natural killer cells), and CD163 (a marker of M2 polarized macrophages). These investigations should soon clarify the correlation between ZEB1/2‐expressing cells and cancer immunity in HNSCC.

The differentiation of cancer cells into those with aggressive traits is needed to initiate invasion into mesenchymal tissues from epithelial tissues. The EMT is thought to be one process that facilitates this differentiation, and it also allows cells to acquire cancer stem‐like properties and drug‐resistant phenotypes (Lambert & Weinberg, 2021). The most well‐known EMT‐TFs in various cancer types are ZEB1 and Snail (Shibue & Weinberg, 2017). Snail promotes cancer cell aggressiveness, including in lung and pancreatic cancer, without altering E‐cadherin expression. Since Snail is identified as a suppressor of the transcription of E‐cadherin in normal cells during the developmental stage, Snail promotes malignant phenotypes in a cancer cell type‐dependent manner (Zheng et al., 2015). In the present study, however, Snail overexpression failed to regulate the expression levels of E‐cadherin and motility/lethality in mice (Figure S1a, and data not shown). In contrast, ZEB1/2 expression was inversely correlated with the epithelial trait in HNSCC. A number of bioinformatic analyses have shown that many cancer cells express ZEB1/2 proteins simultaneously, while several normal cell types do not (Nishimura et al., 2006). In fact, overexpression of ZEB1 increased the expression of endogenous ZEB2 in the present study (Figure 1c). To clarify the mechanisms of this mutual upregulation, we performed several experiments, including promoter analysis and bioinformatics tools, though the underlying molecular mechanisms have not been determined yet. We have previously reported that Ets transcription factors activated by ERK pathways promote transcription of ZEB1 and ZEB2 in breast cancer cells (Sinh et al., 2017), though it is unlikely that overexpression of ZEB1 would activate ERK pathway, due to drastic reduction of cell proliferation (Figure 3a). Another possibility is a double negative feedback loop between ZEB1/2 and the micro RNA‐200 (miR‐200) family (Bracken et al., 2008; Saitoh, 2023). The miRNAs of the miR‐200 family target and repress both ZEB1 and ZEB2, which in turn inhibit the transcription of the miR‐200 family, resulting in a double negative feedback loop controlling ZEB1/ZEB2 and miR‐200 family expression. Thus, it is possible that overexpression of ZEB1 represses the miRNAs of the miR‐200 family and subsequently increases protein levels of ZEB2.

In this study, we generated mouse HNSCC MOC1 cells overexpressing either ZEB1 or ZEB2. The cellular behavior of the two types of cells was similar, except that those overexpressing ZEB2 exhibited greater anchorage‐independent growth. E‐cadherin repression was only detected in cells with silencing of both ZEB1 and ZEB2, but not with either alone. Therefore, it is necessary to develop anti‐cancer drugs that inhibit both ZEB1 and ZEB2 simultaneously, because ZEB1/2 are both deeply involved in the malignant differentiation of cancer cells.

## 4 EXPERIMENTAL PROCEDURES

### 4.1 Cell culture

Mouse mammary epithelial NMuMG cells (Shirakihara et al., 2011) and human TSU and HOC313 cells were described previously (Sakamoto et al., 2021). TSU and HOC313 cell lines were authenticated by short tandem repeat analysis, and then cultured in Dulbecco's modified Eagle medium (Nacalai Tesque, Kyoto, Japan) supplemented with 4.5 g/L glucose, 10% fetal bovine serum (FBS), 50 U/ml penicillin, and 50 μg/ml streptomycin. C57BL/6 MOC cells were kindly provided by Dr. Ravindra Uppaluri (Dana Farber Cancer Institute, Boston, MA) and cultured in MOC medium (Iscove's modified Dulbecco's medium [Nacalai Tesque], Ham's F12 nutrient mixture [Nacalai Tesque], 5% FBS, 50 U/ml penicillin, 50 μg/ml streptomycin, 5 ng/ml epidermal growth factor [Sigma, St. Louis, MO], 400 ng/ml hydrocortisone [Sigma], and 5 mg/ml insulin [Cell Science & Technology, Miyagi, Japan]) (Judd et al., 2012). All cells were cultured at 37°C under a 5% CO2 atmosphere and routinely tested for Mycoplasma.

### 4.2 Reagents and antibodies

Recombinant human TGF‐β1, FGF basic (FGF2), and FGF7 were obtained from R&D Systems (Minneapolis, MN). Rabbit polyclonal anti‐ZEB1 (NBP1‐059870) and anti‐ZEB2 (NBP1‐82991) antibodies were obtained from Novus Biologicals (Littleton, CO). Mouse monoclonal anti‐E‐cadherin (610182), anti‐α‐tubulin (T9026), and anti‐p27 (610241) antibodies were obtained from BD Biosciences (Lexington, KY). Rabbit monoclonal anti‐phospho‐ERK1/2 antibody (#9101S) was obtained from Cell Signaling Technology (Danvers, MA). Goat polyclonal anti‐p21 (SC‐397) antibody was obtained from Santa Cruz Biotechnology (Dallas, TX).

### 4.3 Lentivirus infection

For production of lentiviral vectors, HEK293FT cells were transfected using Lipofectamine 2000 (Thermo Fisher Scientific, Waltham, MA) with pCAG‐HIVgp and pCMV‐VSV‐G‐RSV‐Rev vectors (Horiguchi et al., 2012). The culture media were collected 72–96 h after transfection and used for cell infection.

### 4.4 Immunoblotting

The procedures used for immunoblotting assays were previously described (Shirakihara et al., 2011). Briefly, cells were lysed in lysis buffer (20 mM Tris–HCl [pH 7.5], 150 mM NaCl, 1% Nonidet P‐40, 5 mM EDTA, 1 mM EGTA, and protease and phosphatase inhibitors). Protein concentrations were measured using BCA protein assay reagent (Thermo Fisher Scientific). Harvested proteins were separated by SDS‐PAGE and transferred onto polyvinylidene difluoride membranes. The blots were incubated with antibodies overnight at 4°C. Primary antibodies were diluted to 1:1000. After incubation with secondary horseradish peroxidase‐conjugated anti‐mouse or ‐rabbit IgG (1:10000, Jackson ImmunoResearch Laboratories, West Grove, PA) for 1 h, proteins were visualized using Amersham Biosciences ECL Western blotting detection reagent (GE Healthcare, Little Chalfont, UK). All images were acquired with a Fujifilm LAS‐4000 mini imager and analyzed with Image Reader LAS‐4000 software (Fujifilm, Tokyo, Japan).

### 4.5 Immunofluorescence labeling

The procedures used for immunofluorescence labeling were previously described (Sakamoto et al., 2021). Briefly, cells were fixed in 1:1 acetone‐methanol solution and incubated with antibodies diluted with Blocking One solution (1:500, Nacalai Tesque) for 1 h at room temperature. The cells were then incubated with secondary anti‐rabbit, ‐mouse, or ‐goat IgG (Thermo Fisher Scientific) and TOPRO (Invitrogen Molecular Probes, Eugene, OR) for 1 h. Fluorescence was examined using an Olympus FV1000 (Tokyo, Japan) confocal microscope.

### 4.6 RNA extraction

Total RNA was extracted using the ISOGEN kit (NIPPON GENE, Tokyo, Japan) or the RNeasy Mini Kit with DNase treatment (Qiagen, Venlo, Netherlands), and stored at −80°C until use. The purity of RNA samples was assessed spectrophotometrically by measuring the OD260/280 ratio. Reverse transcription was performed immediately following the quality control assessment.

### 4.7 Conventional PCR

The procedures used for conventional PCR were previously described (Shirakihara et al., 2011). PCR products were separated on 2% agarose gels, stained with ethidium bromide, and visualized using a Printgraph AE‐6932GXES gel detection system (ATTO Corp., Tokyo, Japan). The gene encoding GAPDH was used as an internal control.

### 4.8 RT‐qPCR analysis

The PrimeScript First Strand cDNA synthesis kit (TaKaRa‐Bio, Kusatsu, Japan) was used for synthesizing cDNAs from total RNA. qPCR analyses were performed using the Power SYBR Green PCR Master Mix (Applied Biosystems, Foster City, CA), and the relative expression level of each mRNA was normalized against that of mouse TATA binding protein (TBP) mRNA or human GAPDH mRNA. According to previous reports (Abad et al., 2002; Taylor et al., 2010), a standard curve was ensured by performing two‐fold serial dilution over five points of the most concentrated cDNA sample.

To perform RT‐qPCR analyses, cells were seeded on two wells of a tissue culture plate. mRNAs were extracted from the cells, and each split into three wells of 96‐well plates to measure endogenous mRNA levels by RT‐qPCR. These experiments were repeated at least twice, and representative results are shown in the figures.

### 4.9 RNA interference

Transfection of siRNAs was performed in six‐well tissue culture plates using Lipofectamine RNAiMAX transfection reagent (Thermo Fisher Scientific). The final concentration of siRNA was 5–10 nM. The sequences of siRNAs against human ZEB1/2 used in this study were previously described.

Human ZEB1: top strand, 5′‐CACCGCTACTGGAGATGGCAATTGCCAACAAATTGCCATCTCCAGTAGC‐3′; bottom strand, 5′‐AAAAGCTACTGGAGATGGCAATTTGTTCGCAAATTGCCATCTCCAGTAGC‐3′.

Human ZEB2: top strand, 5′‐CACCGGAGAAAGTACCAGCGGAAACCGAAGTTTCCGCTGGTACTTTCTCC‐3′; bottom strand, 5′‐AAAAGGAGAAAGTACCAGCGGAAACTTCGGTTTCCGCTGGTACTTTCTCC‐3′.

### 4.10 Cell proliferation assay (MTT assay)

After cells were seeded on 96‐well plates, cell viability was measured using Cell Count Reagent SF reagent (Nacalai Tesque), which was added to the media and incubated for 30–60 min, followed by colorimetric measurements at 450 nm.

### 4.11 Invasion assay

Boyden chamber migration assays were conducted using 24‐well cell culture inserts with a transparent PET membrane and an 8.0 μm pore size (BD Falcon, Franklin Lakes, NJ), coated with collagen type I‐C (Nitta Gelatin, Osaka, Japan). After cells were seeded in triplicate on the inserts, cells remaining on the upper faces of the filters were removed using cotton swabs. Cells that invaded the lower surfaces were visually quantitated after fixation in acetone: methanol (1:1) and staining with trypan blue.

### 4.12 Animals

C57BL/6 mice were purchased from CLEA Japan, Inc. (Tokyo, Japan) at 8 weeks of age. All experiments with mice were conducted according to the Guidelines for Proper Conduct of Animal Experiments published by the Science Council of Japan. Protocols were approved by the Animal Care and Use Committee (No. 17‐11) of the University of Yamanashi.

### 4.13 Experimental pulmonary metastasis and HE staining

Mouse MOC cells were suspended in phosphate‐buffered saline and injected intravenously at 1.0 × 10^6^ cells/mouse into the lateral tail vein. For imaging analyses, luciferin was intravenously injected 1–3 weeks later into the retro‐orbital vein of mice anesthetized with isoflurane. The emission intensity was measured using the IVIS Lumina imaging system (SPI Co., Ltd., Tokyo, Japan). Several days later, lung samples were dissected and fixed with 10% neutral buffered formalin (Wako Pure Chemical Industries Ltd., Osaka, Japan), followed by HE staining. Analyses were performed in a blinded manner by pathologists at the Saitama Medical University International Medical Center.

### 4.14 Statistical analyses

Data are presented as means ± SD. Statistical analyses were performed using Student's *t*‐test to compare any two groups, or Tukey's multiple comparison test for multiple comparisons of means against a control group.

## AUTHOR CONTRIBUTIONS

**Arisa Kinouchi:** Conceptualization; data curation; formal analysis; investigation. **Takahiro Jubashi:** Data curation; investigation. **Rikito Tatsuno:** Data curation. **Jiro Ichikawa:** Data curation; supervision. **Kaname Sakamoto:** Data curation; supervision. **Daiju Sakurai:** Supervision. **Tomonori Kawasaki:** Data curation; formal analysis. **Hiroki Ishii:** Funding acquisition; supervision. **Keiji Miyazawa:** Project administration; supervision; writing – review and editing. **Masao Saitoh:** Formal analysis; funding acquisition; project administration; validation; writing – original draft; writing – review and editing.

## FUNDING INFORMATION

This study was supported by the Japan Society for the Promotion of Science (JSPS KAKENHI Grant Number 22H03260, 23K08959, and 21K16828).

## CONFLICT OF INTEREST STATEMENT

The authors declare no conflict of interest.

## ETHICS STATEMENT

All mouse experiments were conducted in accordance with the Guidelines for Proper Conduct of Animal Experiments issued by the Science Council of Japan. Protocols were approved by the Animal Care and Use Committee, University of Yamanashi.

## Supporting information


**DATA S1.** Supporting Information.
