# Supplementary material for: Roles of ZEB1 and ZEB2 in E‐cadherin expression and cell aggressiveness in head and neck cancer
Source: Genes Cells. 2024 Oct 3;29(12):1131–43. doi: 10.1111/gtc.13167 (PMC11609604; doi:10.1111/gtc.13167)
Supplement: Supplementary file 1 — DATA S1. Supporting Information. [file GTC-29-1131-s001.pdf]

Supplementary information

Experimental procedures

Anchorage-independent growth

MOC cells were maintained for 0–48 hours on an ultra-low attachment surface 96-well plate (Corning, Tokyo, Japan). At the end of the assay period, colonies in each well were observed microscopically and statistically evaluated.

Figure S1. (A) MOC1 cells were infected with lentiviruses carrying negative control (NC), Snail, or ZEB1. Infections were independently performed twice (#1 and #2), followed by immunoblot analysis with the indicated antibodies. α-tubulin was used as a loading control. P, parental MOC1 cells (non-infected cells). **(B)** Cells were examined for anchorage-independent growth in soft agar (left in B), and then quantified (right in B). Similar results were obtained in two independent experiments. P values were determined by Tukey's multiple comparison test. *$P < 0.01$.

Figure S1

A

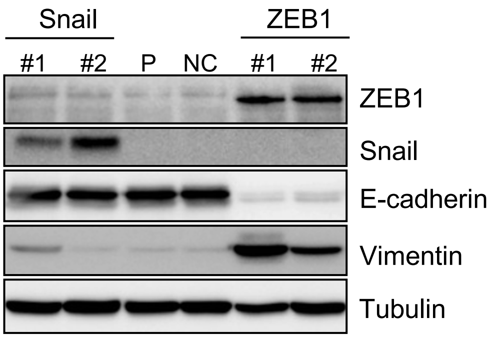

Snail
ZEB1
#1
#2
P
NC
#1
#2
ZEB1
Snail
E-cadherin
Vimentin
Tubulin

B

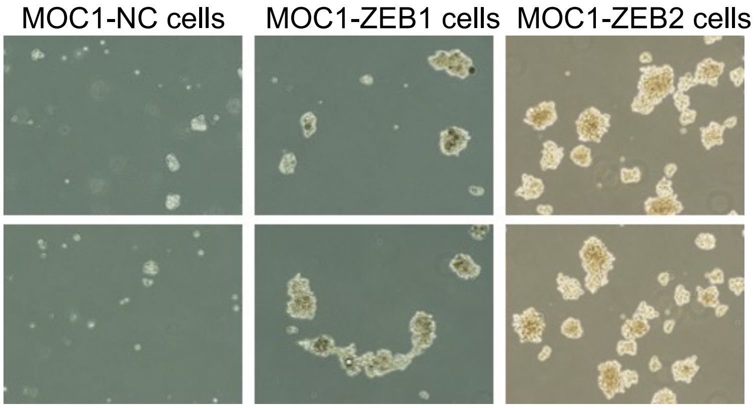

MOC1-NC cells
MOC1-ZEB1 cells
MOC1-ZEB2 cells

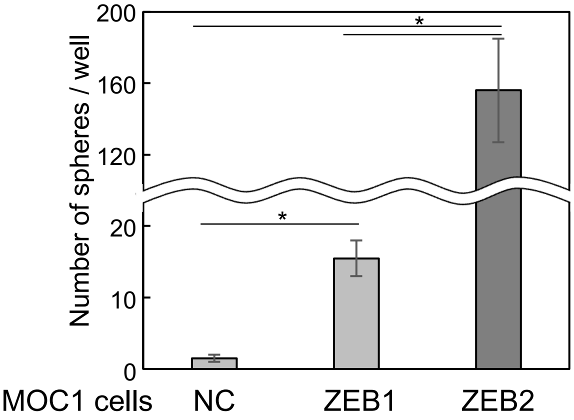

Number of spheres / well
200
160
120
20
10
0
*
*
MOC1 cells
NC
ZEB1
ZEB2
